# Externally-Controlled Systems for Immunotherapy: From Bench to Bedside

**DOI:** 10.3389/fimmu.2020.02044

**Published:** 2020-09-04

**Authors:** María Tristán-Manzano, Pedro Justicia-Lirio, Noelia Maldonado-Pérez, Marina Cortijo-Gutiérrez, Karim Benabdellah, Francisco Martin

**Affiliations:** ^1^Gene and Cell Therapy Unit, Genomic Medicine Department, Pfizer-University of Granada-Junta de Andalucía Centre for Genomics and Oncological Research (GENYO), Granada, Spain; ^2^LentiStem Biotech, Pfizer-University of Granada-Junta de Andalucía Centre for Genomics and Oncological Research (GENYO), Granada, Spain

**Keywords:** immunotherapy, gene therapy, externally controlled, inducible, ATMPs, transgene expression, cancer, autoimmunity

## Abstract

Immunotherapy is a very promising therapeutic approach against cancer that is particularly effective when combined with gene therapy. Immuno-gene therapy approaches have led to the approval of four advanced therapy medicinal products (ATMPs) for the treatment of p53-deficient tumors (Gendicine and Imlygic), refractory acute lymphoblastic leukemia (Kymriah) and large B-cell lymphomas (Yescarta). In spite of these remarkable successes, immunotherapy is still associated with severe side effects for CD19+ malignancies and is inefficient for solid tumors. Controlling transgene expression through an externally administered inductor is envisioned as a potent strategy to improve safety and efficacy of immunotherapy. The aim is to develop smart immunogene therapy-based-ATMPs, which can be controlled by the addition of innocuous drugs or agents, allowing the clinicians to manage the intensity and durability of the therapy. In the present manuscript, we will review the different inducible, versatile and externally controlled gene delivery systems that have been developed and their applications to the field of immunotherapy. We will highlight the advantages and disadvantages of each system and their potential applications in clinics.

## Introduction

Immunotherapy has drastically evolved since the past 30 years, providing diverse approaches for boosting the intrinsic power of the host's immune system to target different diseases, especially cancer. This field includes a broad spectrum of strategies that includes the administration of cytokines, chemokines, monoclonal antibodies, cell lysates, and living cells (1–7) to directly or indirectly boost the immune system to fight cancer or to defuse it for mitigating transplant rejection (8), autoimmune diseases (9), or chronic inflammation (10).

Immunotherapeutic molecules can be delivered systemically or locally into the patients through different systems such as non-viral or viral strategies that can be administered through *in vivo* or *ex vivo* strategies (11). Immuno-gene therapy is a new strategy of immunotherapy that involves genetic modification of cells in order to control immune responses. Some of the most successful immuno-gene therapy applications target tumor cells (12–14) and reduce autoimmune/inflammatory disorders (8, 9, 15).

The re-administration of T cells that are genetically modified to recognize and kill specific cell types (Chimeric Antigen Receptor, CAR-T cells) are particularly successful immunotherapeutic lines to fight refractory tumors (7, 16). Nowadays, Kymriah (Tisagenlecleucel) and Yescarta (Axicabtageneciloleucel, Axi-cel) CAR-T cells became the first two advanced therapy medicinal products (ATMPs) approved in 2017 for the treatment of refractory CD19+ acute lymphoblastic leukemia and aggressive B-cell lymphomas, respectively (17). A third potential ATMP, JCAR017 (Liso-cel) has received the Food and Drug Administration (FDA) breakthrough designation and priority access to medicine program by the European Medicine Agency (EMA) for Relapsed/Refractory Large B-cell Lymphoma (16) and expected to be clinically approved in 2020 (18).

Besides the excellent clinical outcome reported for several immuno-gene therapy approaches, the continuous expression and secretion of potent active molecules [such as IL-12, interferons (IFNs)] can generate adverse clinical events that can lead to life-threatening organ damage and death. This toxicity also limits efficacy, due to the impossibility to reach the appropriate concentrations in target organs. There is therefore a clear necessity to develop fine-tune strategies capable of modulating immune cell activity in order to improve safety and effectiveness of immunotherapies. In this sense, gene therapy field has developed multiple strategies to control the potency and duration of the immune responses by controlling transgene expression.

**Figure 1:**
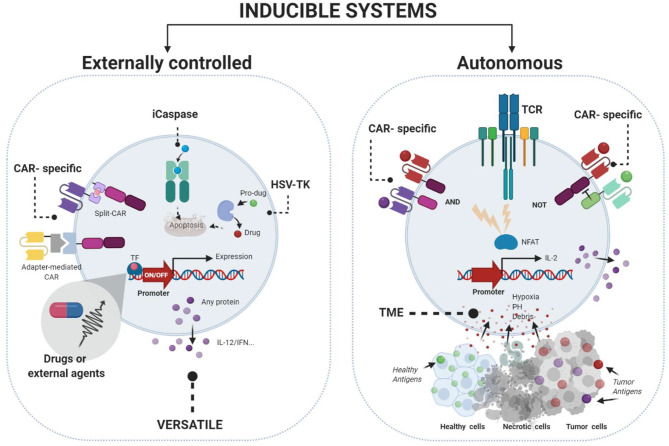
Gene therapy strategies to control immunogene therapy using inducible systems. Externally controlled systems (left) require the addition of an external stimuli (chemical or physical) to modulate the expression of the desire transgene. Autonomous systems (right) are designed to control the expression of the transgene in function of different cellular situations such as inflammation, cytokines, hypoxia, or pH. Figures were created with BioRender.com.

Several autonomous and externally-control strategies for regulating activity in immuno-gene therapy have been developed [reviewed in (19–21)] (Figure 1). First autonomous systems are self-regulated and respond to signals such as stress, inflammation, cytokines, or endogenous hormones. However, those strategies do not allow clinicians to control the intensity and durability of the therapy.

**Figure 2:**
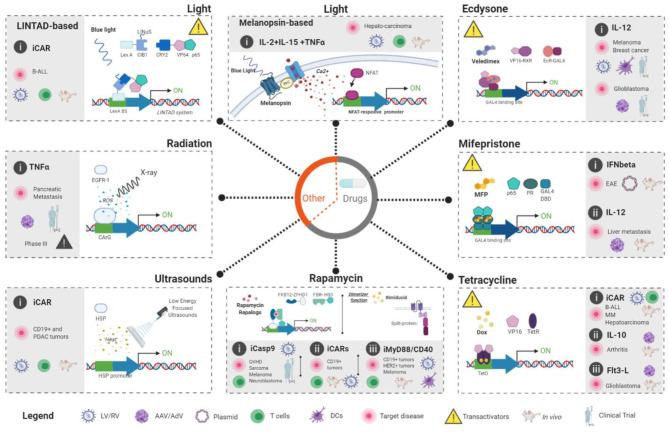
Externally-controlled inducible systems applied to immunogene therapy applications. Two main groups can be established: (1) Drug-based systems (Gray circle in the middle) which include ecdysone, mifepristone, tetracycline, and rapamycin systems. In these systems, clinicians could control the activity of the immunogene therapy through the administration of a drug that will, usually, activate the expression of the desired transgenes. (2) Physical-based systems include two light-based (melanopsin-based and LINTAD), one radiation-based (TNFerade) and one ultrasound-based (FUS-CAR). White area of the each dashed-line square shows the rational of each approach to achieve externally-controlled transgene expression. Key molecular players (inductors and regulatory-proteins) in each system are also indicated. Gray area inside the dashed-line squares indicate published immunogene therapy approaches for each system and the current status; *In vitro* studies (indicated by the absence of a mice or human drawing), *in vivo* studies (indicated by the presence of a mice) and clinical trials (indicated by a drawing of human figure). In addition, the target disease, therapeutic gene, modified cells and vector type are also shown. The legend at the bottom of the figure illustrate the meaning of the different symbols used in the figure. Figures were created with BioRender.com.

On the contrary, remote-controlled systems allow the modulation of activity and associated side effects. Those approaches rely on the co-administration of an inductor, which should fulfill certain characteristics in terms of pharmacokinetics, tolerability and biodistribution (Table 1). There are various systems for controlling gene expression or managing toxicities at different levels (Figure 2). For example, inducible suicide herpes simplex virus tyrosine kinase (HSV-TK) or human thymidylate kinase (TMPK) systems trigger cell death upon a small molecule administration [reviewed in (27)] but are irreversible systems. On the other hand, several systems have been developed to control CAR-T activity (28–31). Despite their clinical potential, they are CAR-specific and not able to control other immuno-gene therapy strategies.

**Table 1:** Characteristics of Dox-inducible Tet-On CARs.

**System**	**Target**	**Delivery**	**Population**	**Doses^**a**^**	***In vivo* induction?**	**Leaking**	**rtTA?**	**ClinicalStage**	**Ref**
Tet-On 3G (TaKaRa Bio)	CD19	Single	Selected	100 ng/ml	Yes (pre-induced)	Yes	Yes	Pre-clinical	(22)
Tet-On (Sangon Biotech)	CD19	Single	Bulk	4 g/ml	No	Yes	Yes	Pre-clinical	(23)
Tet-On 3G(Clontech)	CD38	Dual	Selected	1,000 ng/μl	No	No	Yes	Pre-clinical	(24)
Tet-On 3G (TaKaRa Bio)	CD147	Single	Bulk	1,000 ng/ml	Yes(pre-induced)	Yes	Yes	Pre-clinical	(25, 26)

a *Doses in vitro; Ref, reference*.

In this review, we will focus on externally controlled, reversible (on/off switchable) and versatile inducible systems which can constitute potential tools for improving immunotherapeutic application. We will discuss the benefits and weaknesses of every emerging approach regarding their state of development, safety, on/off dynamics, inductor properties and closeness to clinics.

## Principle of Externally Controlled Systems

Gene therapy provides us a robust, safe and heterogeneous platform of gene transfer for clinical applications. This field has generated a wide range of long-term, stable (or transient, if required) tools, with reduced immunogenicity for modifying immune cells by using non-viral and viral delivery systems.

Although multiple inducible systems have been developed, we will focus on those that are externally controlled, are able to regulate any transgene and are potentially applicable to humans. In order to compare the different versatile available systems for clinical applications, several characteristics must be considered including the inducer properties, vector architecture, and origin, single or dual systems, promoters, target cells, leaking (basal expression in absence of the inducer) and potential risks parameters. For clarity, we will classify the different externally controlled systems on (1) those based on the administration of drugs and (2) those based on the application of physical inductors (light, ultrasounds or irradiation).

### Drug-Inducible Systems

Inducible systems controlled through the administration of drugs are designed to trigger conformational changes on target proteins so they induce (ON-systems) or block (OFF-systems) transcription of the desired transgenes. OFF-systems have the disadvantage of continuous administration of inductor, necessary for silencing transgene expression. Permanent-high levels of antibiotics, for example, can lead to several complications for the patients and have therefore very limited applications in clinics. In this review, we will focus on the ON-systems (Figure 2). These systems require, in general, two key components: (1) a chimeric transcription factor which contains a DNA-binding domain and a drug-binding domain; and (2) a regulated minimal promoter, with very low basal activity, followed by the gene of interest. This promoter includes several copies of a non-natural DNA-binding site in which the chimeric transcription factor binds in the presence of the drug.

#### Tetracycline-Regulated Expression Systems

Tetracycline (Tet)-based gene expression control systems have been established as the systems par excellence for gene induction due to ease of handling, high efficiency and minimal side effects. This system has been designed to have three different configurations: (1) the system based on the original tetracycline repressor, TetR (Tet-ON) (32–34). In these configurations, TetR-binding sites (Tet operator-tetO) are inserted between a constitutive promoter and the gene of interest blocking its activity. The addition of tetracycline or its derivatives [such as doxycycline (Dox)] promotes a conformational change in the TetR that makes it incapable of tetO binding, allowing transcription to proceed. (2) tTA-based systems (Tet-OFF) (35). These systems are based on a chimeric protein formed by the fusion of TetR and a domain of VP16 (derived from *herpes simplex virus type 1*). Contrary to the TetR-based system, here the inducible transgene is placed downstream of a minimal (inactive) promoter harboring tetO sequences. Only if tTA bind to the tetO sequences, will the promoter be active through the activity of the VP16 domain, and will express the transgene. In this configuration, the addition of tetracycline also makes the tTA unable to bind to tetO and transcription stop. (3) rtTA-based systems (Tet-ON) (Figure 2, bottom-right). As mentioned before, Tet-OFF systems have limited applications in clinics. Different groups have therefore designed Tet-On systems based on mutational modifications of tTA in order to allow its binding to the tetO only in the presence of tetracycline. In these new systems, transcription requires the presence of tetracycline, becoming a Tet-ON system. The first Tet-On system (36) based on the rtTA had high leaking, but new developments improved the control of the expression (37–39). However, these systems, as we will discuss in detail, still have important drawbacks for clinical applications due to the presence of transactivators. In this direction, new developments of the original TetR systems have managed to control transgene expression in the absence of transactivators in most cell types analyzed (33, 34), including primary T cells (40). These developments could open new opportunities in the field of immunotherapy.

An important advantage of these systems is that tetracycline and its derivatives, such as doxycycline, have been widely used as antibiotics in humans for decades and have been very well-characterized clinically (41). With 93% of oral absorption efficient, 14–22 h of half-life and deep tissue penetration, including blood-brain barrier (BBB) (Table 2), they are ideal inducing agents for a rapid increase in expression, long-term and rapid decrease of the desired transgene.

**Table 2:** Pharmacokinetics of the small molecules used as inductors for inducible immunotherapy.

**Inductor**	**Type**	**FDA-approved**	**FDA-Dose^**a**^**	**Oral**	**T_**max**_**	**T_**½**_**	**BBB**	**Ref**
Doxycycline	Antibiotic Tetracycline	Yes, for bacterial infections	200 mg/day	Yes	1–3 h	18–22 h	Yes	(41)
Veledimex	Diacylhydrazine	Investigational, Fast Track-FDA, as Inductor	10–20 mg/ml	Yes	2.5–5.5 h	18–27.5 h	Yes	(42)
Mifepristone	Progestational and glucocorticoid antagonist	Yes, abortive, contraceptive	4.5 mg/kg	Yes	1–2 h	15–30 h	Yes	(43)
Rapamycin (Sicrolimus)	Antibiotic macrolide	Yes, as immunosuppressant	2–5 mg/day	Yes	1–6 h	57–68 h	Yes	(44)
Rimiducid (AP1903)	Antibiotic macrolide	Investigational, Orphan-Drug designation	0.4 mg/kg	Yes	N.D	5 h	Yes	(45)

T_max_, peak in blood after administration; T_1/2_, elimination half-life of the drug; BBB, blood-brain barrier; N.D, non-determined; Ref, reference.

a *FDA approved or used in the current clinical trial in adults*.

##### Immunotherapeutic application

*CAR-T cells*. A number of studies utilizing Tet-regulatory systems to regulate CAR expression have been carried out. CAR-T therapy is a promising approach in antitumor therapy, with remarkable results obtained so far in hematological diseases. However, there are important limitations due to uncontrolled responses as a consequence of constitutive expression of the CAR molecules on the surface of T cells. For this reason, a temporary and reversible CAR expression, in which CAR-T cells response can be turned on/off, would be a convenient and eligible solution. Sakemura et al. (22) used the all-in-one pRetroX-TetOne-3G vector in which the CD19CAR-tEGFR sequence was expressed in an inducible manner in primary CD8+ T cells using the rtTA system. CAR+ cells were first selected for obtaining an almost 93% pure population. Maximal CAR expression in SUP-T1 cells was achieved with 100 ng/ml Dox and the expression went down after 20 h of Dox removal, although it did not reach zero in the absence of Dox. Clear differences regarding antitumor efficacy *In vitro* between (Dox+) Tet-CD19 CAR-T cells and (Dox-) Tet-CD19 CAR-T cells were found, but the system exhibited a significant CAR expression in the absence of Dox. For *in vivo* experiments, only Tet-CD19CAR-T cells incubated with Dox prior to inoculation suppressed tumor growth. Following a similar strategy, Gu et al. (23) generated an all-in-one vector expressing the rtTA2S-M2 protein (an improved version of the rtTA) and CD19-CAR (23). In this case, a concentration of 4 μg/ml of Dox was necessary to induce CAR expression 5-fold. Those inducible CAR-T cells also presented better killing of tumor cells in the presence of Dox, although they produced killing also in its absence. The efficiency of the TetOn system has been also tested for multiple myeloma (MM), using CD38 antigen as the target of Dox-regulated CAR T cells (24). Here, the authors used two vectors, the pRetroX-TRE3G vector to control the expression of the CD38-CAR and the pRetroX-TET-On 3G for expression of the rtTA transactivator. CAR-expressing cells were selected by puromycin to obtain a pure population. Maximal tumor lysis *In vitro* was assessed with 1,000 ng/ml and the prompt reversion of the CAR activity was better achieved after a short exposition (24 h) with 10 ng/ml Dox. There are also pre-clinical assays using the Tet-On 3G system in solid tumors, specifically for hepatocellular carcinoma (HCC) treatment (26). Zhang and co-workers constructed the Tet-CD147-CAR lentiviral vector to generate Tet-CD147-CART cells. With a Dox concentration of 1,000 ng/ml CAR expression reached the peak at 24 h and returned to baseline level at 48 h after removal of Dox, but expression never reached zero. CART cells exhibited higher lytic activity in the presence of Dox, but residual lysis as a consequence of CAR leaking was observed. In an HCC mouse model, mice treated with pre-induced (Dox+) Tet-CD147-CART significantly reduced tumor volume and weight compared with those of mice that received (Dox-) Tet-CD147-CART (26), but *in vivo* leaking was noticeable.

*Others immune-gene therapy approaches*. Tet-On systems have also been applied to control cytokine expression in order to boost or control immune responses in a doxycycline-dependent manner. One of the first demonstrations of the potential of this strategy used two adeno-associated vectors (AAVs), one AAV harboring the Tet-responsive promoter driving the expression of interleukin-10 (IL-10) and the other expressing rtTA (46). The authors showed therapeutic efficiency over *In vitro* human rheumatoid synovium from rheumatoid arthritis patients as well as *in vivo* mice model, after intramuscular injection of both AAVs.

In another approach, the group of Dr. Castro developed a combined strategy that used Adenoviral vectors (AdV) to express HSV-TK constitutively and FLT3L in a Dox-dependent manner (47) for the treatment of Glioblastoma multiforme (GBM), a primary malignant brain cancer with very poor prognosis. This strategy aims to induce apoptosis in dividing cells in the presence of ganciclovir, and to stimulate the recruitment of DCs to the site of HSV-TK-mediated tumor killing through Dox-induction of FMS-like tyrosine kinase three ligand (Flt3L). The authors observed significant therapeutic benefits in rat models of GBM after intracranial inoculation of the AdV vectors and after treatment with Dox. Of note, a dose of 300 mg/day Dox was more effective than 200 mg/day equivalent, showing the high Dox concentrations required in this Tet-On system. Interestingly, rats were able to generate adaptive immune responses against the implanted tumors (48). Based on these studies, a clinical trial was approved in 2013 (ClinicalTrials.gov Identifier: NCT01811992) and currently ongoing Phase I (updated on April 2020).

#### Ecdysone-Regulated Expression System

Another interesting system to control gene expression in a rapid, robust, precise, and reversible way are based on the use of steroids-based regulatory domains from insects. Steroids present very interesting properties as inducers of externally-controlled systems: they can penetrate all tissues and are quickly metabolized. The group of R.M. Evans developed the first regulated system based on the ecdysone receptor of *Drosophila melanogaster* to regulate transgene expression on mammalian cells (49). Different versions of these systems have been published since with different success in different cell types and tissues (50, 51). Of all the systems, the RheoSwitch (52) has been the most successful, with applications even in clinical trials.

The RheoSwitch Therapeutic System® (RTS) consists of a series of inter-dependent functional components for gene induction (Figure 2, top-right): (1) two transcription factors (VP16-RXR and Gal4-EcR), (2) an inducible promoter and (3) an activating small molecule ligand. The first factor arises from the fusion between the ligand binding domains of a chimeric RXR and the transcriptional activation domain of VP16 of HSV1, that acts as a co-activation partner. The second consists of a DNA-binding domain of the yeast transcription factor Gal4 fused with the hinge domains of the mutated ecdysone receptor (EcR) of the Spruce budworm (*Choristoneurafumiferana*), where the ligand is bound. To achieve regulation, both proteins must be constitutively expressed. The addition of the ligand promotes the stabilization of the heterodimeric complex which binds the responsive-promoter through Gal4 and leads to transcriptional activation thanks to the VP16 domain. In the absence of an inducer, the complex is destabilized and transcription is blocked.

The RTS system has been clinically validated for the control of IL-12 expression through clinical trials (53). Previous studies using IL-12 were based on the use of strong constitutive promoters, such as CMV or EF1-α to achieve high expression levels. However, IL-12 plays crucial roles in naive T cells differentiation into cytotoxic T-lymphocytes (CTLs) via IFN-γ production. It does need therefore a clear control in order to achieve the desired therapeutic benefits minimizing side effects.

Different groups have also investigated the most appropriate ligand to be used in clinical settings (54). Ecdysteroids are contained in vegetables thus its safety for humans is well-proven. Veledimex is a synthetic analog of ecdysone used as the ligand of the RheoSwitch system and is currently under investigational in the Fast-track line of FDA due to its pharmacokinetics features (42, 55) (Table 2).

##### Immunotherapeutic applications

The VP16-RXR and Gal4-EcR sequences was adapted into an AdV vector to express IL-12 under the control of the RTS [reviewed in (56)]. Using this rationale, two strategies were followed: (1) to transduce dendritic cells (DCs) *ex vivo* and introduce them into the patients, and (2) to introduce the Ad-vector *in vivo* (56). In the first strategy, a complete tumor regression was reached in a subcutaneous B16F0 melanoma model by delivering mIL-12-DCs intratumorally (57, 58). Using the second strategy, between 73 and 90% of tumor regression was obtained using the melanoma model and tested successfully against other tumoral models (56, 59). In all the cases, IL-12 increased DCs life, generated a high infiltration into the tumors of cytotoxic CD4+ and CD8+ T cells producing high levels ofIFNγ. Based on these data, the first-in-human clinical trial was approved that used externally-regulated gene therapy intervention (NCT00815607). This first study aimed to analyze safety, regulation of the IL-12, tolerance, response rate, and immunological effects. Patients enrolled received 5 × 10^7^ DCs transduced with Ad-RTS-hIL12 and oral administration of inducer ranging from 0.6 to 200 mg. A second phase I clinical trial was also approved using the second strategy. Patients were injected with 1 × 10^12^ Ad-RTS-IL-12 particles into accessible lesions in combination with oral inducer administration. Patients included had stage III-IVmelanoma (NCT01397708) and metastatic breast cancer (NCT01703754, NCT02423902). Although only a minority of the patients achieved a partial regression, a veledimex dose-dependent increment of mRNA IL-12 intratumorally as well as serum IFNγ levels were manifested (60). Unfortunately, several patients experienced serious toxic effects but were rapidly solved after veledimex discontinuation (60, 61). In another phase-I study targeting Glioblastoma (NCT02026271), the authors showed a significant improvement in patient's survival (55). In this study, several patients experienced severe adverse events (CRS or neurological-related) that were quickly controlled after suspension of veledimex uptake. Today, there are four open clinical trials to evaluate the intratumoral injection of Ad-RTS-hIl-12 and activated with oral veledimex (20 mg/day during 15 days) as a therapy to treat patients with recurrent or progressive glioblastoma (alone or combined with anti-PDL1 monoclonal antibody (mAb), NCT04006119, NCT03679754, NCT03330197, NCT03636477).

#### Mifepristone-Regulated Expression System

The first development of Mifepristone (MFP)-based systems (62) took advantage of the modular nature of functional domains of steroid receptors. The authors generated a Mifepristone-responsive regulator (pGL-VP) by fusing the ligand-binding domain of the human progesterone receptor, the DNA-binding domain of yeast GAL4 protein and the VP16 transactivation domain of the HSV protein. They showed that this chimeric protein was able to promote transcription of minimal promoters containing GAL4-binding sites after administration of MFP *In vitro* and *in vivo*. Importantly for these systems, the MFP (RU486) concentration required for transgene activation is lower than that required for antagonizing progesterone action.

A later development consisted in a chimeric regulator, GLp65 composed of a mutated ligand-binding domain (LBD) of the human progesterone receptor, the DNA-binding domain of yeast GAL4 protein and the activator domain (AD) from the human p65 protein, part of the nuclear factor kappa B complex (63). This system was commercially named as the GeneSwitch^TM^ (GS) platform (64). GS needs two expression cassettes: the first one expressed constitutively (normally through the CMV promoter) the GLp65 transactivator protein, and the second cassette includes the inducible promoter, which contains at least four sequences for GAL4 binding, and the gene of interest. When MFP is present and binds to the LBD, a conformational change allows the GLp65 transactivator to dimerize and binds to the GAL4-promoter, activating transcription through the p65 domain (Figure 2, middle-right). The elimination of the VP16-domain from the system reduced expression levels but also reduced leaking, improved safety and reduced immunogenicity.

MFP is a clinically approved drug, with anti-progestin and anti-glucocorticoid properties (65). The long-term use of this drug in both females and males is under current investigation in phase III for psychotic depression (66) but MFP appeared safe and well-tolerated at the doses required to activate transcription (Table 2). However, the potential side effects as a glucocorticoid antagonist should be further characterized. Its progesterone antagonistic activity could be a problem on human T cells, which present progesterone receptors in the membrane, and T cell proliferation was inhibited after 5 mM of MPF (43, 67). Altogether, suggest that dose-escalation for future clinical application should be carefully validated.

Different GS system has been developed for inducible gene therapy approaches to fight liver cancer (68–70), as well as for the treatment of neurological diseases (71, 72).

Another important aspect to consider of inducible systems is the alterations provoked by the constitutive expression of the chimeric regulators. Reboredo et al. (73) analyzed the effect of GLp65 and rtTA2(S)-M2 in the liver's transcriptomics. They found that while rtTA2 expression induced alterations in 69 genes, GLp65 caused an altered expression of 1,059, although functional analysis showed only mild alterations.

##### Immunotherapeutic applications

GS system has also been applied to regulate the expression of potent cytokines such as IL-12 with the aim to control its activity while keeping their therapeutic potential. In an elegant study, Wang et al. developed a strategy to achieve hepatic-specific expression of IL-12 that also responded to the control of MFP. The authors developed an AdV harboring the sequences for GAL4 binding into a hepatic-specific promoter driving the expression of IL-12 (6). Direct administration of these AdVs enabled controlled hIL-12 expression in the liver for more than 48 weeks when MFP was administered every 24 h. In addition, this system achieved complete tumor regression in an aggressive model of liver metastases *in vivo*. Whereas, using a specific-liver promoter seems to be useful for preventing immunogenicity, the IL-12 production in the liver was associated with a moderate inflammatory reaction opens the possibility that higher doses of AdV-MFP could induce-IL-12-related severe inflammation.

In a different approach, MFP-GS was used to express IFN-beta for the treatment of a murine model of multiple sclerosis, experimental autoinflammatory disease (EAE) (74). In that model, a single intramuscular administration of the inducible mIFNβ vector delivered as DNA plasmid was sufficient to decrease significantly the onset of disease. The procedure was well-tolerated and the overall severity of the disease scores was reduced in the presence of MFP (74).

#### Rapamycin-Regulated Expression System

Rapamycin-regulated system is a human platform designed by Rivera et al. (75, 76) that is based on the interaction between two cytosolic proteins that only dimerizes in the presence of rapamycin. FK506 binding protein (FKBP12) is a 12 kDa cytosolic protein and FKBP12-rapamycin-binding protein (FRB) is a 11 kDa domain derived from mammalian target of rapamycin (mTOR). The original system contained three copies of FKB12 fused to a DNA-binding domain (zinc finger homeodomain transcriptional factor 1, ZFHD1) composing the DBD and FRB was fused to the DNA activation domain (AD) of Nuclear Factor Kappa B p65 subunit, driven expression of the gene of interest in a three-plasmid system. Transgene expression was induced after a 24 h incubation with 10 nM of rapamycin (75) but induction failed when DBD was incorporated in a retroviral vector (77). A new and more potent AD domain, called SH3, containing sequences from human heat shock factor one (HSF1) and p65, overcomes that problem even with only one copy of FKB12 in a single vector and placing the target gene cassette in reverse orientation achieves no leaking. In this case, 1 μM rapamycin or analog AP1903 was necessary for maximal induction (Figure 2, bottom-middle).

Rapamycin (Sirolimus) is a macrolide antibiotic with potent immunosuppressant activity used for allograft rejection in renal and cardiac transplantation (45). This immunosuppressive action occurs by targeting calcineurin and IL-2 production in T cells by Rapamacyin-FKB12 and inhibiting mTOR, thus affecting cell proliferation and metabolism via Rapamycin-FBR. In order to improve safety, a mutation in the FKBP domain was generated (FKBP12-F36V) (78) to allow the design of novel rapalogs (AP1903/AP20187) that bind the mutated but not the wild-type FKBP protein. Therefore, AP1903 (Rimiducid) is a safe and well-tolerated drug that can be administered up to 1 mg/kg (44, 79) (Table 2).

##### Immunotherapeutic applications

Since these systems are based in human-derived components, they present minimal immunogenicity and have been efficiently adapted to immunotherapy (Table 3). In addition, the inductor is able to cross the BBB (84) and required low concentrations (78, 84) (Table 2).

**Table 3:** Systems for controlling transgene expression applied to immunotherapy.

**System**	**Inductor**	**Gene**	**Model/Disease**	**Product**	**Administration**	**Clinical stage**	**Ref**
Tet-On 3G (TaKaRa Bio)	Dox	CAR-CD19	CD19+ Raji cells (Burkitt's lymphoma)	All-in-one RV-T cells	Cells^a^: intravenously Inductor: pre-induced *ex vivo*+ oral	Pre-clinical	(22)
Tet-On (Sangon Biotech)	Dox	CAR-CD19	CD19+ Raji cells (Burkitt's lymphoma)	All-in-one LV-T cells	*In vivo* experiments were not conducted	*In vitro*	(23)
Tet-On 3G (Clontech)	Dox	CAR-CD38	CD38+ cell lines (Multiple myeloma)	Dual system RV-T cells	*in vivo* experiments were not conducted	*In vitro*	(24)
Tet-On 3G (TaKaRa Bio)	Dox	CAR-CD147	CD147+ cells (Hepatocellular carcinoma)	All-in-one LV-T cells	Cells^a^: intratumoral Inductor: pre-induced *ex vivo*	Pre-clinical	(26)
Tet-On	Dox	IL-10	DBA1 mice (Rheumatoidarthritis)	All-in-one AAV vp	Vector^b^: intramuscularly Inductor: oral	Pre-clinical	(46)
Tet-On	Dox	FLT3L	Glioblastoma multiforme	All-in-one Ad	Vector^b^: intracranial Inductor: oral	Phase I	(47)
RheoSwitch (RTS)	Veledimex	IL-12	Stage III or IV melanoma	All-in-one Ad-DCs	Cells^a^: intratumoral Inductor: oral	Phase I	(57)
RheoSwitch (RTS)	Veledimex	IL-12	Stage III-IV melanoma Metastatic breast cancer	All-in-one AdV	Vector^b^: accessible lesions Inductor: oral	Phase I/II	(56)
Gene Switch	MFP	IL-12	MC-38 mice (Livermetastases)	All-in-one AdV	Vector^b^: intravenous Inductor: intraperitoneal	Pre-clinical	(68)
Gene Switch	MFP	IFN-β	EAE mice (multiple sclerosis)	DNA plasmid	Plasmid^c^: intramuscular Inductor: subcutaneous	Pre-clinical	(74)
Light- pNFAT	Blue light	IL-2, IL-15, TNF-α	SK-HEP-1 mouse (Hepatocellular carcinoma)	All-in-one LV-T cells	Cells^a^: subcutaneous Inductor: externally applied	Pre-clinical	(80)
LINTAD	Blue light	CAR-CD19	CD19+ Nalm6+ mice (B-lymphoblastic leukemia)	Dual system LV-T cells	Cells^a^: subcutaneous Inductor: externally applied	Pre-clinical	(81)
TNFerade	Radiation	TFN-a	Metastatic pancreatic cancer	All-in-one Deficient AdV	Vector^b^: intratumor Inductor: externally applied	Phase III	(82)
FUS-CAR	Ultra-sounds	CAR-CD19	CD19+ Nalm6+ cells (B-lymphoblastic leukemia) PC3 cells (Prostatecancer)	Dual system LV-T cells	Cells^a^: subcutaneous Inductor: externally applied	Pre-clinical	(83)

a Ex vivo transduction.

b in vivo transduction.

c *Direct plasmid injection Dox, doxycycline; MFP, mifepristone; CAR, chimeric antigen receptor; IL-10, interleukin 10; FLT3L, FMS-like tyrosine kinase 3 ligand; IL-12, interleukin 12; IFN-β, interferonβ; IL-2, interleukin 2; IL-15, interleukin 15; TNF-α, tumor necrosis factor α; EAE, experimental autoimmune encephalomyelitis; RV, retroviral vector; LV, lentiviral vector; AAV, Adeno-associated vector; vp, viral particles; Ad, adenoviral vector; Ref, reference*.

One of the most important uses of this system has been adapted to induce the activation of the proapoptotic enzyme caspase 9 (85), initially adapted to kill tumor cells, it did translate soon for suicide and irreversible T-cell depletion (86) to treat GVHD (BPX-015, Phase I/II). iCasp9 or CaspaCIDE is based on the homodimerization of mutated FKB12 fused to the signaling domain of caspase 9 after the treatment of AP1903/Rimiducid. This system can eliminate 85 to 95% of circulating CD3+ T cells within 30 min [NCT01494103 (84)]. A phase I trial had demonstrated long-term-persistence of transduced T cells (up to 3.6 years) without compromising proliferation. However, a single clone of iCasp9-transduced T cells caused a delayed CRS in one patient that developed *de novo* Epstein–Barr virus-associated post-transplant lymphoproliferative disease (EBV-PTLD), being unresponsiveness to Rimiducid (87).

iCasp9 have been also included in TCR-restricted (88) and CAR-T cell therapies as a safety measure (89). iCasp9 showed efficient clearance in anti-CD19 CAR-T cells co-expressing IL-15 (90) and in third generation anti-CD20 CAR-T cells, where the 90% of engineered T cells were depleted *in vivo* in only 12 h (91). GD2-specific and iCasp9-expressing CAR (GD2-iCAR) T cells have reached clinical trials against advanced melanoma (CARPETS, ACTRN12613000198729), neuroblastoma (GRAIN, NCT01822652), sarcoma (VEGAS, NCT01953900), and other GD2+ solid tumors (NCT02107963). Of note, while iCasp9 can rapidly reverse toxicity, sacrifices the long-term antitumor efficacy. Stavruo et al. (78) have exploited the same Caspase 9 strategy in CAR19-T cells but using original FRB/FKBP system to generate homodimers of Casp9 after rapamycin addition (RapaCasp9), which is indeed a clinically-drug approved, exhibiting a similar response of rapaCasp9 to iCasp9 at 1 nM (78).

Another elegant FKBP/FRBmut system specific for CARs, are the “ON-switch” CARs (28), where the CAR structure is split into two chimeric polypeptides: CAR-I encloses the antigen recognition domain, transmembrane and 4-1BB costimulatory domain and CAR-II harbors the main CD3zeta-ITAMS signaling domain. Both chimeric proteins are fused to intracellular FKBP/FRBmut. Only when AP21967 was administered, an anti-tumoral effect was observed in mice, but due to its shorter life, another heterodimer system would be desirable for a more-suitable future clinical application.

In a different configuration but with a similar idea, the dimerizing agent–regulated immunoreceptor complex (DARIC)-T cells is also composed of two CARs (92): the CAR-I is composed by the ScFv-FRB-TM domains and the CAR-II by the FKPB-TM-41BB-CD3ζ domains. In both CARs, the FKBP/FRBmut domains are located extracellularly. DARIC-T cells also allow the application of a plugin for targeting another antigen (a subunit of the ScFv and FRB). In addition, Tacrolimus, which has a high affinity for FKBP12, can be used as a rapamycin competitor, which could be interesting as a safe method for reducing severe CRS or persistent neurotoxicity (92).

Controlling the ScFv presentation at the cell surface after AP21967 addition is another FKB16/FRBmut design, where the FRB and FKBP12 domains were placed between the CD8a hinge and the scFv domains, modulating the cytotoxic properties of this “transient” CAR-T cell (93).

Rapamycin-based systems have also been developed to activate immune cells such as dendritic cells (DC) or T cells, regulating the synergy of TLR/IL1R (through MyD88) and CD40 signaling within the context of an immunological synapse. In this case, rimiducid-inducible MyD88 and CD40 (iMC) system is composed by aTIR domain-deleted version of MyD88 fused to tandem copies of the modified FKBP12V36 and a myristylation-targeting sequence for membrane anchoring, whereas the same fusion structure was used for the cytosolic domain of CD40. Autologous iMCs-DCs showed a strong antitumoral effect *in vivo* (94, 95).

More recently, this iMC strategy has been applied for the CAR-T field against HER2+ solid tumors. In the presence of Rimiducid, T cells expressing HER2–CARζ and this FKBP12 iMyD88/CD40/FKBP12 system exhibited potent antitumor activity in pre-clinical models, allowing their remote-control post-infusion (96), becoming a very promising platform. Duong et al. (97) engineered CAR-T cells with this Rimiducid iMC-signaling system for CAR T cell activation in combination with the rapamycin-induced caspase-9-based safety switch (iRC9) for controlling potential risks. This dual-switch (DS) system generated higher CAR-T cell expansion in a drug-dependent manner while triggering apoptosis to avoid severe toxicities if required (97). However, escalation doses of Rim to *in vivo/*clinical application should be carefully evaluated since 100x more of the Rim dose for activate iMC counterpart (1 nM), could also trigger the iRC9 system *in vitro*.

#### Future Drug-Inducible Systems for Immunogene Therapy Applications

Here, we will briefly describe systems that fulfill the above criteria of versatile, reversible and inducible system but have not been used yet for immunotherapeutic applications.

##### Antibiotics

Other antibiotic-based systems found in different bacterial strains have been modified and adapted to control gene expression in mammalian cells such as streptogramin (PipOFF/PipON) and macrolide (EON/EOFF) based gene regulation systems (98). Those four systems have been tested *In vitro* using different cell lines and different transgenes, obtaining a fast (<24 h) and great induction (until 100-fold) of transgene and low leaking (98, 99). Both the macrolide and the streptogramin antibiotic families present interesting clinical properties, such as excellent bioavailability, optimal pharmacokinetics, and human compatibility (100, 101).

Another system that has not been applied yet to immunotherapy approaches is the system based on the original tetracycline repressor, TetR (32–34). These systems have several advantages over the traditional Dox-based system that use transactivators such as the absence of toxicity, the low leaking, and the low Dox requirements.

##### Quorum Sensing

A chimeric transcription factor controlled by an acylated homoserine lactone (AHL), getting up to 1000-fold induction and low basal transcription in different human cell lines have been developed (102). However, AHL signaling molecules can influence the behavior of eukaryotic cells and tissues and it is unknown its pharmacodynamics *in vivo* (103, 104). Looking ahead, it is possible to engineer other transcription factors from different bacterial species and develop inducer compounds with improved characteristics.

### Physically-Induced Systems

#### Light-Based Systems (Optogenetics-Based)

Optogenetics rely on light-sensitive proteins that have a physiological role of regulating the behavior of living cells. Most optogenetic tools are based on light-sensitive ion channels, but there are also other types of molecules able to respond to light, such as enzymes and protein interaction modules (105–110). This variety of tools has opened the opportunity of modulating gene transcription and to use it for gene therapy applications (111–113). Transgene inducible-systems based in Optogenetic are a very promising approach due to their high spatial-temporal control capacity (114) compared to other systems and because light can be applied locally without affecting other organs. However, the low penetrance of blue light may be a limiting factor for future clinical application. Two main optogenetic strategies have been used in immunotherapy: Melanopsin-based (calcineurin-NFAT-based) and biLINuS-based (nuclear translocation induced by light).

The Melanopsin-based system (Figure 2, top-middle) relies on the ability of this protein to induce calcium influx under blue light illumination. Intracellular Ca^++^ increment activates calcineurin that triggers the nuclear translocation of NFAT (111), a transcription factor involved in the expression of multiple genes related to effective immune responses (115). For the system to achieve light-response into target cells we need to express the melanopsin and introduce an expression cassette harboring an NFAT-responsive promoter (Figure 2) (111). Once all the components are into the target cell, light will activate Ca^++^ influx through the melanopsin that is expressed in target cells. This Ca^++^ influx initiates a signaling cascade that leads to NFAT-nuclear translocation, activation of NFAT-promoter and expression of the desired genes.

In the biLINuS system (Figure 2, top-left), the light will expose the NLS motif to cause nuclear translocation of the complex (generally harboring transcriptional activators) required for transcriptional activation. These systems are based in the light-inducible nuclear localization signal (LINuS) from the LOV2 domain of Avena sativa phototropin 1 (ASP-1), a small tag that can be added to different proteins and cell types (116). In particular, the LINuS system has been used in combination with the blue light–based CRY2-CIB1 (used for blue light-dependent transgene expression) (117) but that had a high background. Huang et al. (81) developed the LINTAD gene activation system that rely in two chimeric proteins and a light responsive promoter (Figure 2): (1) The LexA-CIB1-biLINuS (LCB) protein combines the CRY2-CIB1 pair with the LOV2 domain reducing non-specific CRY2/CIB1 dimerization, (2) the CV protein contains the NLS from CRY2PHR (*Arabidopsis CRY2* photolase homology region) and a strong VPR transcription activator (a tripartite VP64-p65-Rta), and (3) the light-inducible promoter harbors several LexA-binding sequence (LexA BS) and a minimal promoter that require the presence of transactivators to be active. The LCB remains in the cytoplasm, while the CV remains in the nucleus. When stimulated by blue light, biLINuS in the LCB is activated, exposing the NLS motif to cause nuclear translocation of the SCB. At the same time, the CRY2PHR domain in CV is activated by blue light and can bind to the CIB1 domain of LCB with high affinity. Therefore, the LCB-CV complex is directed to the LexA BS in the reporter cassette so that the VPR is very close to the minimal promoter, which triggers transcription of the target reporter gene. This would generate a strong activation of the gene by stimulating blue light with a high signal-to-noise ratio.

##### Immunotherapeutic applications

Following the two main strategies described above, optogenetics have pursued two main strategies for immunotherapy: to increase the expression of NFAT-targeted genes (mainly cytokines), key regulators of T cell function (80, 112, 115, 118) and to induce CAR expression through the biLINuS system (80, 116).

*Expression of NFAT-targeted genes*. Based on the melanopsin-based system, Zhao et al. (80) designed a light control system for the inducible expression of three molecules: IL-2, IL-15, and TNFα. The system relies on the expression of melanopsin on T cells in order to change T-cell functions via the NFAT-calcium pathway. The T cells must also be modified to contain a NFAT-responsive promoter expressing the desired factors. The author included all these components into T cells using Lentiviral vectors and generated T cells that express increased amounts of IL-2, IL-15, and TNFα after blue light stimulation. In this system, light activates melanopsin to induce Ca2+ input triggering NFAT nuclear translocation and cytokine NFAT-dependent gene expression of IL-2, IL-15, and TNFα. This enhances the tumor killing activity of T cells, a crucial factor for immunotherapy to be effective for solid tumors. The system was tested on pan-T cells, and checked for up-regulation of IL-2, IL-15, and TNFα by messenger RNA and protein after light stimulation during 12 h of continuous exposition. Maximum reporter expression (determined in 293T) was achieved 1-6 h after point-light stimulation and reached basal levels at 48 h, but not light-kinetics were performed over primary T cells. This group demonstrated *in vivo* light-controlled antitumor efficacy in a subcutaneous model of hepatocellular carcinoma, SK-HEP1 in NSG mice. Engineered T cells were introduced intratumorally, and blue LED light applied for 7 days, showed significant tumor regression (80).

*Light-inducible CAR-T cells*. As described above, Huang et al. (81) generated a blue-light transgene inducible system (LINTAD) with low background that allowed the generation of a light-inducible CAR. Indeed, Huang et al. demonstrated that they could regulate the expression of several genes (including CAR) by blue light administration *In vitro* and *in vivo*. The authors engineered T cells to express constitutively the LCB and CV light-responsive proteins and to integrate a light-inducible promoter to express a CAR targeting CD19. These light-inducible CAR-T cells were able to lyse Nalm-6 cells (CD19+) 7.3-fold more efficiently after providing light. Importantly, the system also worked *in vivo* since significant differences in tumor regression was observed under blue-light conditions (1 s-pulse every 30 s during 12 h) with respect to the dark state (81).

#### Radiation-Controlled System

Radio-genetic therapy takes advantage of radiation and gene therapy for cancer applications, controlling by radiation the expression of a therapeutic gene. Ionizing radiation (IR) induces DNA damage such as double strand breaks (DSBs) and reactive oxygen species (ROS) that activate certain signaling pathways of mammalian cells (SAPK/JNK or DNA-PK) in order to offer an early (expression of transcription factors) and late response against that dangerous stress. IR induces the expression of TNF-alpha, IL-1 and other growth factors and metabolic enzymes for DNA repair, mutagenesis, apoptosis, and proliferation. Those inducible promoters constitute interesting tools such as transcription factors AP-1, NFkB, or Early growth response-1 (EGFR) that respond to ROS thanks to the upstream presence of the CArG box, a sequence composed by CC-(A+T rich)-6GG. Hallahan and his team developed the first AdV that controls TNF-alpha under six radio-inducible CArG boxes of *EGFR-1* gene designed as TNFerade (119) and showed a tumor regression in several xenograft models (see below) (Figure 2, left-middle).

Other groups have developed other artificial X-ray inducible promoters in order to overcome the cell type-dependent limitations of natural physiological promoters (120, 121) for prostate cancer. A combination of different cis-elements based on NF-kB, AP-1, NF-Y and CArG, among others, produced a candidate promoter able to regulate luciferase with a peak at 6–10 h post 10 Gy radiation, exhibiting certain antitumor efficacy but with high leaking.

Radiation systems should be further improved in order to achieve minimal basal activity if considered for other clinical applications or methods of administration and obtain more-sensitive promoters, thus 10 Gy dose is relatively high for radiotherapy (fractions of 1.8−2Gy per day are normally used in adults) (122). A treatment of several low-fractionated doses of radiation will be desirably employed for minimizing severe damage, that would depend on the tumor-type and kinetics of the therapeutic gene.

##### Immunotherapeutic applications

TNFerade, developed by GenVec, is a deficient AdV designed for intratumoral administration and that regulated human TNF-α under a radiation-inducible promoter, approved for phase I clinical trials by FDA in 2000 (82). TNFerade has been used over a dosage range of 4 × 10^7^-1 × 10^12^ vp with 30–70 Gy synergistic radiation for several types of solid cancer such as pancreas, esophagus, rectum, breast, lung, skin, head-neck carcinoma, and soft tissue sarcoma, demonstrating improved overall and progression-free survival of cancer patients in phase I (82). Unfortunately, phase III clinical trial for locally metastatic pancreatic cancer was discontinued in 2011, when TNFerade did not increase patient survival in comparison to standard treatment (123). Instead of its proven safety, other limitations of TNFerade include its administration, only feasible to accessible cancers; a spillover out of tumor neighborhood can be a serious issue and moreover, a possible immune response against adenovector may accelerate the metabolism of TNFerade and therapy became ineffective (82).

#### Ultrasounds

Focused ultrasounds (FUs) can be also used as inductor for externally control of gene expression, penetrating with a depth of centimeters into tissues. Therapeutic FUs-controlled by Magnetic Resonance Imaging (MRI) have been applied into clinics for vasodilation, neuromodulation, heat-ablation of tumors and as an adjuvant therapy for drug, gene delivery (124) and tumor vaccination *in situ* (125). Low-energy focused ultrasounds (LO-FUs, with normal intensities of 0.1–2 W/cm^2^ and frequencies of 0.5–3 MHz) generate rapid oscillating pressures that lead to non-invasive hyperthermia. In response, mechanical and thermal stresses are manifested transiently without killing the cells and several genes are upregulated such as heat-shock proteins that translocate from cytoplasm to cell surface (125). Based on this response, the use of heat-shock protein's (HSP) promoters have been used for local control gene expression in several models (126) but not for immunotherapy. However, this rationale has been recently applied for CAR-T regulation induced by low-intensity FUs (83) (Figure 2, bottom-left). In this case, a HSP promoter drives the transgene expression upon FUs stimulation controlled by Magnetic Resonance Imaging (MRI) thermometry. This is a reversible system that generates oscillatory patterns of expression after repeated stimulation of 10 min-FUs every 48 h in T cells, thus preventing short- and long-term side effects.

However, several authors have developed acoustogenetics systems for maintaining a sustained expression, making it irreversible but avoiding FUs pulses to minimize cell death and facilitate treatment application through the adaptation of the Cre-LoxP system in a dual approach.

##### Immunotherapeutic applications

Based in the FU-based Cre-LoxP system, Wu et al. (83) developed a two-vector system in which the HSP promoter drives de expression of Cre recombinase in one inducible LV vector whereas a second vector allow the CAR production after the excision of a LoxP-flanked “STOP” cassette (83). *In vitro* CAR expression in primary T cells was detected 24 h later after a 15 min-pulsed FU, reaching 43°C. Minimal cell death with pulsed-FUS was observed when compared to continuous stimulation during the same period. In addition, this dual system was able to control the cytotoxic potential of inducible CAR-T cells against subcutaneous models of CD19 + Nalm6 cells and human prostate cancer PC3 cells, treated with three pulses of 5 min-FUs, whereas FUs alone did not had tumoricidal effect.

This specific design overcomes the continuous requirement of inductor, whose application is not as easy as a drug-based inducer, but making it irreversible and not allowing a safer control of CAR-T against CRS and neurotoxicity in CD19+ leukemia. Moreover, HSP are translocated to membranes after LO-FUs exposition in cancer cells. This HSP complexes can activate natural killer cells, being interpreted as danger signals for DC activation and cross-presented for generated immunity (125). Whereas, this response is very desirable for tumor treatment for the generation of immune priming and activation of the TME, should be further studied in primary T cells, in order to analyze the worst scenario of a non-desirable immunogenicity against CAR-T cells that can compromise the persistence of the therapy.

## Discussion

Immuno-gene therapy has revolutionized the treatment of chemo/radio-refractory cancers, sometimes reaching the frontline. As mentioned above, adoptive cell transfer based on the use of CAR-T cells has enabled the rescue of many patients by boosting their immune system. Novartis' Kymriah has achieved a complete tumor regression of 60% and Kite's Yescarta, between 36 and 54% (16). But this is not risk-free, due to severe side effects and rapid exhaustion of T-cells by uncontrolled expression of CAR (127). On the other hand, these treatments have been focused on aggressive leukemias and lymphomas, since their efficacy in solid tumors is very low, which requires alternative approaches to convert a “cold” to a “hot” battlefront into the tumor microenvironment (TME) (31, 128).

There is a lot of work to do yet, and this is where expression control systems by external stimuli become important. Being able to externally control the expression of different molecules of interest (CAR, immune-checkpoint inhibitors, cytokines.) opens new ways of re-orienting immunotherapies toward safety and efficacy (19–21). Controlling the expression of CAR will allow reducing CRS, on-target/off-tumor effects and T-cell exhaustion. In addition, controlling other molecules of interest will increase the effectiveness in the treatment of resistant lymphomas and solid tumors. In fact, CAR has already been co-expressed with interleukins (IL-12 or IL-18) and/or with antibodies against PD-1/CTLA-4, in a constitutive way (129–132). Controlling the expression of these molecules may not only enhance therapy in solid tumors, but also avoid the devastating side effects of continued expression in patients.

In this review, we have focused on inducible systems that meet four characteristics: (1) the expression of the transgene can be externally-controlled, (2) they are reversible systems, they can be turn on and off multiple times. (3) They are versatile, they can control the expression of any transgene and (4) they have been adapted for immunotherapy. We found four different systems that meet these criteria: drug, light, radiation and ultrasound-based systems.

Below we discuss different aspects that must be considered when applying these systems for immunotherapy strategies: the properties of the inductor (penetrance, viability, stability toxicity, immunogenicity, etc.), the characteristics of the elements required to achieve external regulation (toxicity, secondary effects, immunogenic, etc) and finally, the ability to deliver all components into the desired cells or tissues.

### Inductor Properties

#### Bioavailability

Doxycycline, veledimex and mifepristone have a similar persistence of 20 h (T_1/2_ in Table 2), good to achieve continuous stimulation for 1 day and also for a quick elimination upon drug removal. However, rapamycin has a T_1/2_ of ~60 h, which makes the system “off” slower, while rimiducid, with a T_1/2_ of 5 h, has a very fast switch-off, but require frequent drug administrations. Due to their small size and polar properties, all these inductors are able to cross the BBB.

#### Side Effects

In general, mid-high and continuous doses of most of the inductors mentioned before are not ideal. Antibiotics can generate a serious antibiotic resistance. Tet systems that have been used to express the CAR require a Dox concentration (*In vitro*) between 10–1,000 ng/ml, that overlaps with the doses required to kill bacteria and will therefore generate resistance if prolonged or intermittent exposure. The same could apply for rapamycin-regulated systems. Therefore, long-term studies should be performed to investigate in both cases how microbiota can also be altered.

Another important aspect to consider is the effect of the inductor in immunomodulation. In fact, most of the drugs used as inductors have an immunomodulatory effect; Rapamycin can efficiently immunosuppress activation and proliferation of T cells (133). To avoid this problem, rapamycin analogs such as Rimiducid can be used as inductors of the mutated-rapamycin system, thus reducing the affinity for the natural FKBP domain (78). Doxycycline has an anti-inflammatory effect by targeting NF-kB (134) and could therefore interfere with the immunotherapy strategy. It is therefore important to reduce the Dox concentration not only to avoid antibiotic resistance, but also to reduce the possibility of immune-suppression. In this direction, new Dox-regulated systems based on the original TetR could be an important resource due to the low Dox concentrations required for activity (34, 40). Finally, MFP acts as an antagonist of glucocorticoid receptors in non-reproductive cells, also present in T cell membrane, and can therefore exert a role blocking the T- activation (67).

#### Physical Inducers

Compared to drugs, the use of light or FUs as inductors has advantages and disadvantages. Firstly, light is, in theory, an ideal induction agent, since it allows the most precise spatial-temporal regulation, simply by applying light to a localized body region the induction takes place in that area. However, the system relies on blue light which is ideal for safety reasons but limits the penetration into the tissue. Probably, red or infrared light systems will overcome the penetration concern, but at the moment are less efficient and require additional cofactors plus photo-activatable domains. Radiation has emerged as an alternative induction agent, although its safety must be considered since actual systems use 10 Gy radiation, 3–4 times higher than the doses used for radiotherapy. Finally, the use of short-pulse pattern stimulation of FUS minimizes the side effects of ultrasound exposure, such as hyperthermia or induction of severe immune responses. In fact, the reversible FUS-inducible system may prevent the on-target/off-tumor toxicity of canonical CAR-T therapy. This occurs because T-cells that leave the tumor environment do not receive FUS again (since the induction is localized), so they will gradually lose membrane CAR molecules. Ultrasound pulses also allow precise temporal control of gene expression. Of note, different companies are manufacturing wearable ultrasonic emitter patches that could be useful for induction through this system (83).

### Characteristics of the Elements Required to Achieve External Regulation

For an externally controlled system to be successful, the most important part is probably the characteristics of the different components that are required. Simplicity as well as low toxicity and immunogenicity are the three most important characteristics to compile after, of course, a high inducibility and a low leaking.

#### Toxicity of the Components

Although, in general, the expression of new proteins into a cell can alter its intrinsic properties, most of the proteins are well-tolerated and allow the cells to fulfill the functions that the scientist expect from them. However, chimeric proteins harboring transactivators, present in several of the described systems, exhibit a great capacity to recruit and sequester different transcription factors [e.g., TATA-binding protein (135)]. The majority of the Tet-based systems, the mifepristone-based, the light-inducible LINTAD system and the ecdysone-based ATMP require either viral or human chimeric transactivators (VP16, VP64, p65). In-deep studies have been performed in this regard with the Tet-platforms showing that the TetR-VP16 protein may be toxic by altering cell physiology and binding to pseudo-TetO sites which can activate undesired genes (136, 137). Furthermore, several studies (138–140) have shown a misinterpretation of the data due to the high toxicity of transactivators. In this direction, Benabdellah et al. (33, 34) have developed the first and only all-in-one, transactivator-free Dox-inducible system based on the original bacterial system. Studies are undergoing to investigate the advantage of these systems for immunotherapy applications.

#### Immunogenicity of the Components

The immunogenicity of all the elements required to achieve the transgene induction is another key point in the success of the system for clinical applications. In general, human proteins are going to be less immunogenic than viral and bacterial components, although in some cases this could not always be the case. The only system that is 100% human is the platform based on rapamycin. All other systems include yeast, bacterial, or viral components that are generally highly immunogenic and to which healthy individuals will mount an immune response. In particular, the immunogenicity of Tet-transactivator-dependent systems has been studied in detail finding that both cellular and humoral responses are mounted when using viral (141, 142) and non-viral (143–145) systems for *in vivo* delivery. Therefore, final strategies using these systems should include strategies that achieve immune tolerization of these components if a durable effect is desired. In fact, different approaches are being investigated to avoid these responses (146), including different routes of administration (147) that successfully achieved long term regulation in animal models.

## Author Contributions

MT-M and PJ-L designed this study, wrote the first draft of the manuscript, first screening of papers, and revised the manuscript. NM-P screened papers and contributed to the writing and revision of the manuscript. MC-G and KB wrote and revised the manuscript. FM designed this study, contributed with critical revision, writing of the manuscript, and paper screening. All authors approved the final version of the manuscript.

## Conflict of Interest

PJ-L was contractually linked to LentiStem Biotech, a Spin Off company that have the license for Lent-On-plus technology. The remaining authors declare that the research was conducted in the absence of any commercial or financial relationships that could be construed as a potential conflict of interest.
